# False positive PSMA PET for tumor remnants in the irradiated prostate and other interpretation pitfalls in a prospective multi-center trial

**DOI:** 10.1007/s00259-020-04945-1

**Published:** 2020-08-17

**Authors:** Wolfgang P. Fendler, Jeremie Calais, Matthias Eiber, Jeffrey P. Simko, John Kurhanewicz, Romelyn Delos Santos, Felix Y. Feng, Robert E. Reiter, Matthew B. Rettig, Nicholas G. Nickols, Amar U. Kishan, PSMA PET Reader Group, Okamoto Shozo, Louise Emmett, Helle D. Zacho, Harun Ilhan, Christoph Rischpler, Axel Wetter, Heiko Schoder, Irene A. Burger, Roger Slavik, Peter R. Carroll, Courtney Lawhn-Heath, Ken Herrmann, Johannes Czernin, Thomas A. Hope

**Affiliations:** 1 Department of Molecular and Medical Pharmacology, Ahmanson Translational Imaging Division, University of California Los Angeles, Los Angeles, CA USA; 2 Department of Nuclear Medicine, University of Duisburg-Essen, Essen, Germany; 3 Department of Nuclear Medicine, Klinikum rechts der Isar, Technical University of Munich, Munich, Germany; 4 Department of Anatomic Pathology and Department of Urology, University of California San Francisco, San Francisco, CA USA; 5 Department of Urology, University of California San Francisco, San Francisco, CA USA; 6 Department of Urology, UCLA Medical Center, University of California Los Angeles, Los Angeles, CA USA; 7 Department of Medicine, Division of Hematology/Oncology, University of California Los Angeles, Los Angeles, CA USA; 8 Department of Radiation Oncology, VA Greater Los Angeles Healthcare System, University of California Los Angeles, Los Angeles, CA USA; 9 Department of Radiation Oncology, University of California Los Angeles, Los Angeles, CA USA; 10 Departments of Radiology and Biomedical Imaging and Pharmaceutical Chemistry, University of California San Francisco, San Francisco, CA USA

**Keywords:** PSMA, PET, Pitfall, Recurrence, Interpretation, Radiotherapy

## Abstract

**Purpose:**

Readers need to be informed about potential pitfalls of [^68^Ga]Ga-PSMA-11 PET interpretation.

**Methods:**

Here we report [^68^Ga]Ga-PSMA-11 PET findings discordant with the histopathology/composite reference standard in a recently published prospective trial on 635 patients with biochemically recurrent prostate cancer.

**Results:**

Consensus reads were false positive in 20 regions of 17/217 (8%) patients with lesion validation. Majority of the false positive interpretations (13 of 20, 65%) occurred in the context of suspected prostate (bed) relapse (T) after radiotherapy (*n* = 11); other false positive findings were noted for prostate bed post prostatectomy (T, *n* = 2), pelvic nodes (N, *n* = 2), or extra pelvic lesions (M, *n* = 5). Major sources of false positive findings were PSMA-expressing residual adenocarcinoma with marked post-radiotherapy treatment effect. False negative interpretation occurred in 8 regions of 6/79 (8%) patients with histopathology validation, including prostate (bed) (*n* = 5), pelvic nodes (*n* = 1), and extra pelvic lesions (*n* = 2). Lesions were missed mostly due to small metastases or adjacent bladder/urine uptake.

**Conclusion:**

[^68^Ga]Ga-PSMA-11 PET at biochemical recurrence resulted in less than 10% false positive interpretations. Post-radiotherapy prostate uptake was a major source of [^68^Ga]Ga-PSMA-11 PET false positivity. In few cases, PET correctly detects residual PSMA expression post-radiotherapy, originating however from treated, benign tissue or potentially indolent tumor remnants.

**Trial registration number:**

ClinicalTrials.gov Identifiers: NCT02940262 and NCT03353740.

**Electronic supplementary material:**

The online version of this article (10.1007/s00259-020-04945-1) contains supplementary material, which is available to authorized users.

## Introduction

Positron-emission-tomography (PET) using [^68^Ga]Gallium-labeled ligands of the prostate-specific membrane antigen (PSMA) localizes recurrent prostate cancer with high accuracy and significant impact on management as demonstrated in several retrospective reports and a recent prospective study [1–4]. At low serum PSA levels, detection rate and reproducibility are superior compared with approved ^18^F-fluciclovine PET [5]. Consequently, clinical [^68^Ga]Ga-PSMA-11 PET application has expanded rapidly, and more recently prospective PET-guided interventional trials aimed at improved survival have been initiated ([6] and NCT03525288). Overall, false [^68^Ga]Ga-PSMA-11 PET interpretations by trained physicians occur in less than 10% of cases [4]. However, physicians need to be informed about potential pitfalls in order to improve quality of their interpretations. Here we report details for [^68^Ga]Ga-PSMA-11 PET findings that were discordant with the reference standard in a recently published prospective trial [4]. We aim to characterize sources of [^68^Ga]Ga-PSMA-11 PET misinterpretation and potential limitations of the reference standard.

## Material and methods

Enrollment criteria, imaging, and lesion validation protocols have been reported previously [4]. In brief, inclusion criteria were histopathology proven prostate adenocarcinoma and biochemical recurrence. Biochemical recurrence was defined as PSA ≥ 0.2 ng/mL more than 6 weeks after prostatectomy or PSA ≥ 2 ng/mL rises above nadir following radiation therapy (ASTRO-Phoenix consensus definition). Patients were enrolled irrespective of prior imaging findings.

Patients underwent PET/CT (*n* = 443, 70%) or PET/MRI (*n* = 192, 30%) based on availability and contraindications. Imaging parameters are given in Supplemental Table 1.

Interpretation and validation criteria were reported previously [4]. In brief, cases were assigned to nine off-site readers (three readers per dataset) that were not involved in study design and data acquisition. All readers underwent training based on a previous dataset [7]. Readers were provided whole body PET (attenuation corrected and non-corrected), whole body post-contrast CT, or whole-body post-gadolinium T1 and pelvic T2 MRI. Most recent PSA level and type of primary therapy (prostatectomy versus radiation therapy) were disclosed; however, readers were blind to all other information. The presence of prostate cancer (positive versus negative) as well as visual PSMA expression was recorded. Consensus was determined by majority vote.

PET positive findings were validated as true or false positive. Regions, negative on [^68^Ga]Ga-PSMA-11 PET, but with subsequently confirmed prostate cancer by histopathology, were considered false negative. True negative was not defined. Descriptive statistics are provided.

## Results

**Fig. 1:**
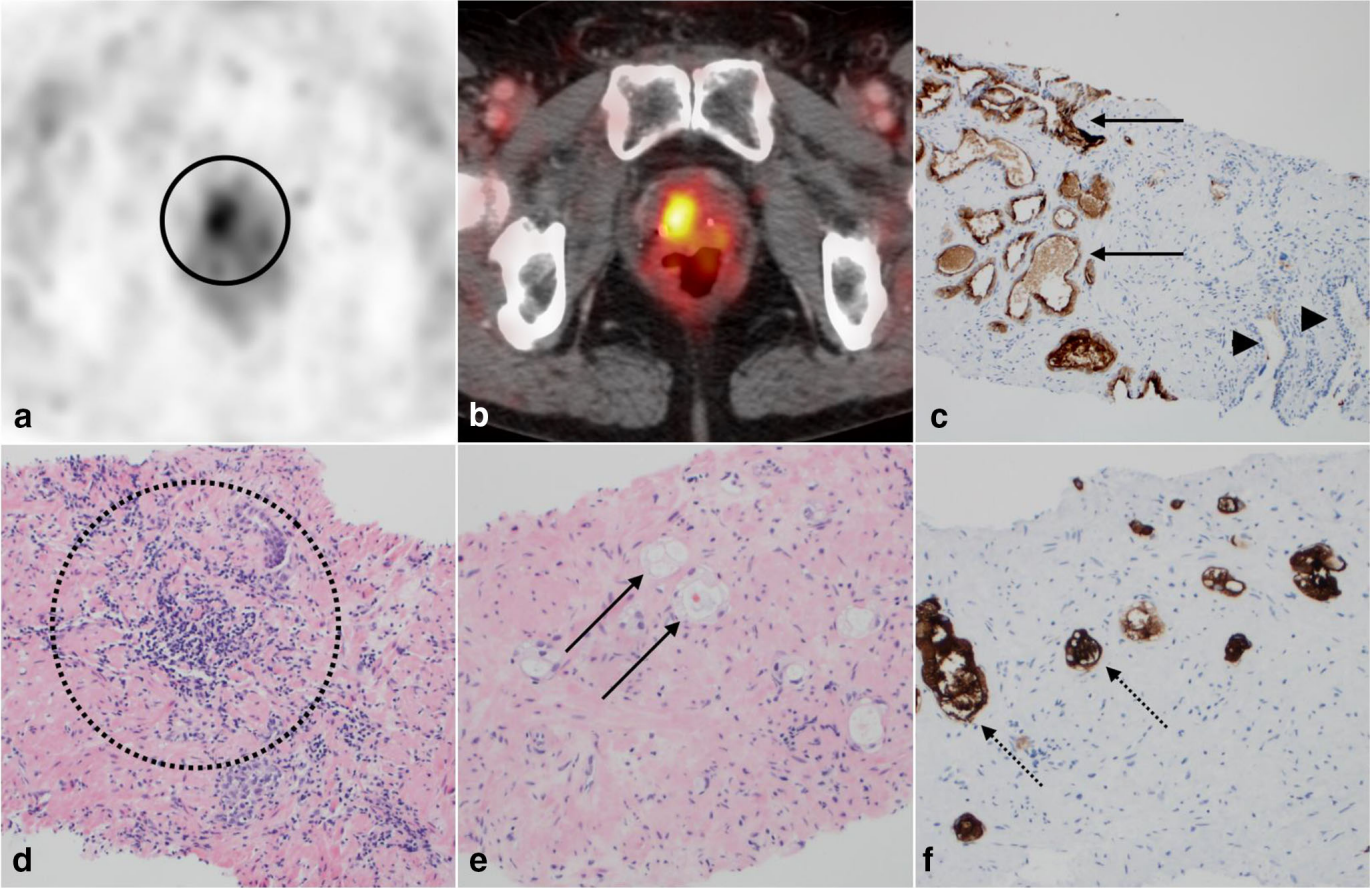
70-year-old man post-radiotherapy 10 years prior, who demonstrates focal [^68^Ga]Ga-PSMA-11 uptake in the right medial prostate (**a**, circle; **b**) (PSA 2.6 ng/mL, CT FP 3). Trans-anal ultrasound guided core needle biopsy demonstrated no evidence of viable tumor. The specimen in the region of the focal uptake (SUV_max_ 9.1) demonstrated marked radiation changes in residual benign glands (**d**, dotted circle), cancer with treatment effect including balloon cells (**e**, black arrows), and cells with marked PSMA expression (**c** and **f**, black arrows). Adjacent benign glands did not demonstrate PSMA expression (**c**, black arrow heads)

**Fig. 2:**
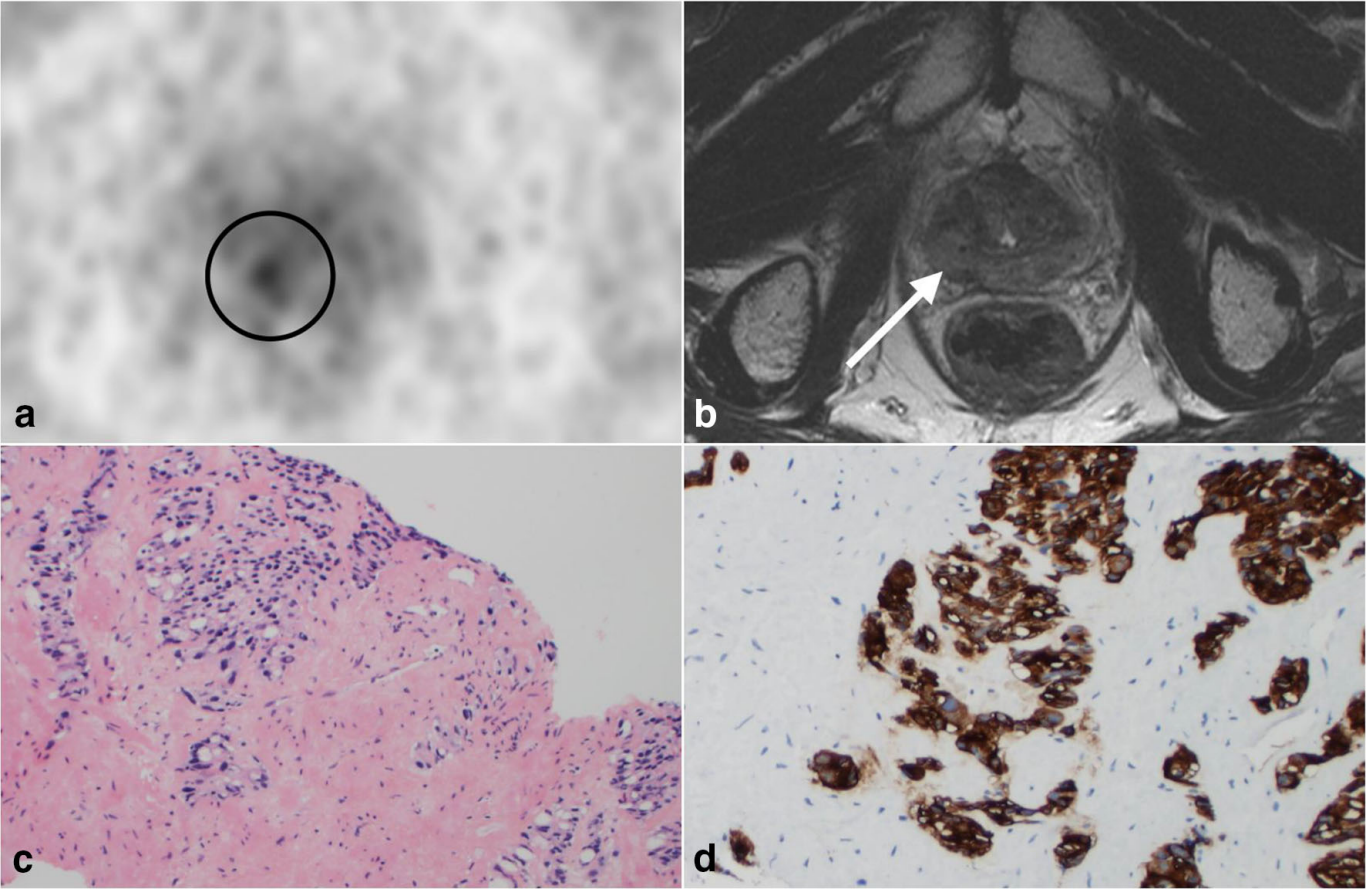
74-year-old man post-radiotherapy performed 10 months prior with biochemical recurrence who had focal uptake on the right peripheral zone of the prostate (**a**, circle) (PSA 7.2 ng/mL, MRI FP 7). Neither T2 weighted imaging (**b**, arrow), dynamic contrast enhanced (DCE) imaging or diffusion weighted imaging demonstrated a focal lesion to correlate with the focal uptake (SUV_max_ 12.5) seen on [^68^Ga]Ga-PSMA-11. Trans-anal ultrasound guided core needle biopsy was obtained from the region of uptake. Pathology demonstrates tumor cells with significant treatment effect (**c**) and marked PSMA expression (**d**). There was no evidence of PSMA expression in vasculature

Characteristics of the entire cohort have been reported previously [4]. Details for false positive findings on a region basis are given in Table 1. Summary images are shown in Supplemental Figure 1. Consensus reads were false positive in 20 regions of 17/217 (8%) patients with lesion validation. Eleven of 20 (55%) false positive cases were documented for suspected relapse in the prostate after radiotherapy. These 11 cases had lesions within the prostate that demonstrated residual PSMA expression months to years after radiotherapy despite benign tissue or cell appearance consistent with successful treatment response. Examples of residual PSMA expression are illustrated in Figs. 1 and 2. Of note, consensus reads were true positive in 36 of 47 (77%) cases with suspected relapse in the prostate post radiotherapy.

**Table 1:** [^68^Ga]Ga-PSMA-11 PET/CT and PET/MRI false positive findings. Findings are illustrated in Supplemental Figure 1A (CT) and B (MRI). miTNM stage in accordance with PROMISE [8]. Abbreviations: FP false positive, Tr prostate bed, N1 pelvic nodes, M1 extrapelvic, brachy brachytherapy, RT radiotherapy, RP prostatectomy

Case No	PSA (ng/mL)	Time from initial therapy (years)	Subregion	N false readers	CT/MRI	Visual PET uptake (SUV_max_)	Validation	Details
CT FP 1	4.3	12	Tr	3	No lesion	Intermediate (5.4)	Biopsy	Post-brachy, “benign“
CT FP 2	2.4	6	Tr	3	No lesion	Low (3.1)	Biopsy	Post-brachy, “benign with fibrosis“
CT FP 3	2.6	10	Tr	3	No lesion	Intermediate (9.1)	Biopsy	Post-RT, “adenocarcinoma with marked treatment effect“
CT FP 4	22.6	3	Tr	2	No lesion	Low (2.9)	Biopsy	Post-RT, “benign with extensive fibrosis“
CT FP 5	10.8	19	Tr	3	No lesion	Intermediate (9.4)	PSA (44% decrease)	Post-RT, bladder/urine uptake
CT FP 6	0.9	1	Tr	2	Contrast enhancement	Intermediate (10.2)	Surgery	Post-RP, inflammation/abscess
CT FP 7	0.8	4	N1 (pelvic right)	2	No lesion	Low (3.4)	Imaging	Ureter/urine uptake
CT FP 8	2.7	3	N1 (mesorectal)	3	No lesion	Intermediate (7.6)	Imaging	3 mm node, size change below threshold
CT FP 9	3.1	9	M1a (retroperitoneal)	3	No lesion	Intermediate (5.2)	Imaging	4 mm node, size change below threshold
CT FP 10	374	22	M1a (mediastinal)	3	No lesion	High (31.0)	Imaging	6 mm node, size change below threshold
CT FP 11	0.7	3	M1b (pelvis)	3	No lesion	Intermediate (11.1)	Imaging	No lesion on follow-up imaging
MRI FP 1	2.7	8	Tr	3	No lesion	Intermediate (11.1)	Biopsy	Post-brachy, “benign“
MRI FP 2	3.8	10	Tr	2	No lesion	High (33.7)	Biopsy	Post-brachy, “benign“
MRI FP 3	4.6	1	Tr	3	8 mm	High (13.0)	Biopsy	Post-brachy, “adenocarcinoma with treatment effect“
MRI FP 4	6.4	1	Tr	2	No lesion	Intermediate (5.4)	Biopsy	Post-RT, “benign“
MRI FP 5	1.2	7	Tr	2	10 mm	Intermediate (16.9)	Biopsy	Post-RT, “benign“
MRI FP 6	4.1	6	Tr	2	5 mm	Intermediate (6.4)	Biopsy	Post-RP, “benign“
MRI FP 7	7.2	1	Tr	3	No lesion	High (12.5)	Biopsy	Post-RT, “adenocarcinoma with marked treatment effect“
MRI FP 8	4.7	8	M1c (lung)	3	No lesion	High (13.0)	Biopsy	Bronchogenic cyst with glands
MRI FP 9	5.2	17	M1c (lung)	3	67 mm	Intermediate (9.1)	Surgery	Lung cancer

**Fig. 3:**
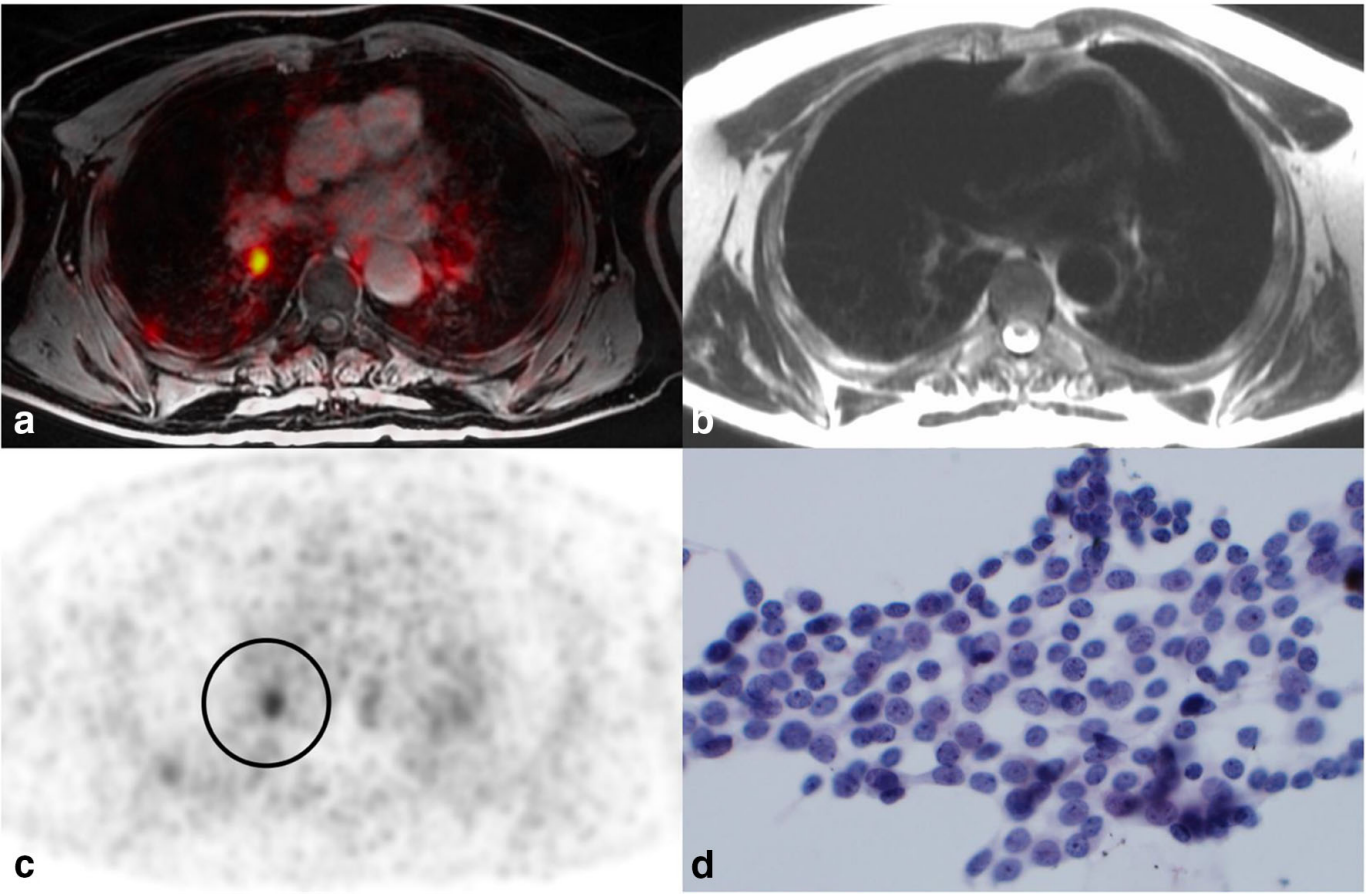
68-year-old man post-radiotherapy therapy with focal uptake (SUV_max_ 13.0) noted in the right hilum on [^68^Ga]Ga-PSMA-11 PET (**a** and **c**, circle) (PSA 4.7 ng/mL, MRI FP 8). Single shot fast spin echo (**b**) does not visualize the lesion highlighting limitations of PET/MRI for characterization of small lung findings. Transbronchial biopsy demonstrates cuboidal epithelium (**d**) with adjacent prominent glands typical for a bronchogenic cyst.

In two cases, uptake in the prostate bed after prostatectomy led to false positive PET interpretation. For one of the two patients, subsequent surgery confirmed the presence of an inter-sphincteric abscess. In four cases, small pelvic or retroperitoneal nodes were associated with false positive findings. Uptake in the lung led to false positive interpretation for prostate cancer in 2 cases (1 patient with lung cancer and 1 patient with bronchogenic cyst; Fig. 3). Visual PET uptake was intermediate to low in 15 (75%) and high in 5 of 20 (25%) false positive lesions according to PROMISE [8].

Eight cases of false negative interpretation were documented. Cause of the false negative interpretation was adjacent urine/bladder/rectum uptake in 4 of 8 (50%) and small size of metastases in 3 of 8 (38%). Of note, no scatter artifact from high urine activity was noted. Details for false negative findings on a region basis are given in Supplemental Table 2. Summary images are shown in Supplemental Figure 2. One lung lesion demonstrated no uptake on PET/CT and was false negative potentially due to partial volume effect and/or respiratory movement. One bone biopsy was performed, which confirmed prostate cancer (true positive).

## Discussion

Our recent prospective trial reports 84 to 92% [^68^Ga]Ga-PSMA-11 PET positive predictive value and 75% overall detection rate for localization of recurrent prostate cancer [4]. [^68^Ga]Ga-PSMA-11 PET is an imaging test for PSMA, expressed at high level by most prostate cancer lesions. Biodistribution of the radiotracer and PSMA expression level of prostate cancer and normal tissues may lead to false clinical interpretations. Here we present details for false PET interpretations of the blinded consensus reads.

[^68^Ga]Ga-PSMA-11 PET at biochemical recurrence resulted in less than 10% false positive interpretations. About two-thirds of all documented false positive interpretations occurred in the context of suspected recurrence in the prostate post-radiotherapy. Following radiation therapy, cancer and prostate tissue undergo considerable molecular and histologic change over time. Effects vary with dose and duration of therapy, interval from onset, and addition of systemic treatment [9]. Prostate cancer regresses slowly, and complete histologic resolution may take several years [10, 11]. Typically, post-radiotherapy biopsy results are rated positive for prostate cancer (accompanied with Gleason Scoring), severe treatment effect, or negative for prostate cancer [12]. In a previous report severe treatment effect and negative biopsy were associated with similar risk for biochemical failure, distant metastases, and cause specific mortality [13]. Risk for both categories was significantly lower when compared with positive biopsy [13]. Residual adenocarcinoma with severe treatment effect was seen on biopsy for 3 of 11 false positive post-radiotherapy prostate specimens in our trial. Despite severe treatment effect immunohistochemistry demonstrated high PSMA expression of tumor cells and adjacent glands (Figs. 1 and 2). It was shown previously that molecular features such as PSA expression or molecular weight keratin can be retained in adenocarcinoma despite marked response to radiation therapy [10].

Rising PSA may originate from lesions outside the prostate, not detected by [^68^Ga]Ga-PSMA-11 PET. However, residual prostate cancer with severe treatment effect, even without conversion into progressive disease, is another possible source of PSA [10]. In observational trials, disease-free survival in patients with residual prostate cancer with severe treatment effect versus negative biopsy was similar [13, 14]. This suggests that PSMA expression within the irradiated prostate, detected by PET, does not necessarily indicate active disease. However, clinical significance of PSMA-expressing remnants post-radiotherapy has yet to be assessed in the setting of biochemical failure. Of note, PET consensus reads were true positive in 36 of 47 (77%) cases with suspected relapse in the prostate post radiotherapy.

In general, patients in a BCR setting are at particular risk for early disease progression, providing a rationale for physicians to offer salvage treatment after primary radiotherapy [15]. However, in previous studies post-radiotherapy residual cancer with severe treatment effect was not associated with progression or poor survival [13, 14]. In patients with isolated and late prostate recurrence, observation remains a favorable management option, and potentially morbid salvage therapy should be considered with caution in the absence of histopathologic verification or extra-prostatic progression [16].

Accuracy of biopsy for the diagnosis of prostate cancer is somewhat limited as demonstrated by previously reported discordance with whole-gland pathology [17]. Sample interpretation, which requires accurate separation of carcinoma from its many mimics and discrimination of treatment effects in normal tissue from recurrent or persistent carcinoma, is difficult [9]. With the availability of highly sensitive [^68^Ga]Ga-PSMA-11 PET imaging, we anticipate that the frequency of imaging detected potential local failures after prostate radiotherapy will increase. This in turn will increase the number of post-radiation biopsies requiring careful pathologic evaluation prior to initiation of salvage therapy.

[^68^Ga]Ga-PSMA-11 PET/MRI interpretation resulted in two false positive lung findings. One patient demonstrated focal tracer uptake of right upper lobe lung cancer. Focal uptake was interpreted as prostate cancer lung metastasis by all three readers. This interpretation accounts for a false positive finding with respect to prostate cancer detection; however, subsequent diagnosis of lung cancer considerably altered oncologic management. Another patient demonstrated focal uptake in a bronchogenic cyst with prominent peribronchial glands (Fig. 3). Of note, peribronchial glands are similar to salivary gland epithelium with known high physiologic radioligand accumulation. Similar mechanism in peribronchial glands may explain focal [^68^Ga]Ga-PSMA-11 uptake here.

In three cases, sub-centimeter nodes demonstrated intense focal uptake on PET; however, lymph node size did not change significantly under systemic therapy to confirm nodal involvement in accordance with reference standard criteria. Intrinsic limitations of validation criteria, especially limited size change in small lesions, have been discussed previously [4]. On the other hand, [^68^Ga]Ga-PSMA-11 PET reaches detection limits for small nodal metastases, especially close to areas of high physiologic uptake. In line with previous reports, false negative findings occurred for small size metastases or adjacent bladder/urine uptake [18, 19]. Additional CT urography demonstrated value in identification of [^68^Ga]Ga-PSMA-11 PET findings adjacent to the urinary system [20].

## Conclusion

A prospective multi-center trial with blinded reads and lesion validation revealed false positive [^68^Ga]Ga-PSMA-11 PET in 20 regions of 17/217 (8%) patients [4]. Faint to moderate [^68^Ga]Ga-PSMA-11 uptake in the prostate post-radiotherapy was a major source of false positive findings. Uptake was noted in PSMA-expressing benign tissue or tumor remnants with successful treatment response by histopathology. Previous studies report limited prognostic relevance of these remnants underlining the importance of additional pathologic evaluation [13, 14]. Other pitfalls were inflammation for false positive, and urine activity or small size metastases for false negative [^68^Ga]Ga-PSMA-11 PET.

### Electronic supplementary material

ESM 1 (DOCX 641 kb)

## Appendix

PSMA PET Reader Group: Okamoto Shozo, MD^8^; Louise Emmett, MD^9^; Helle D. Zacho, MD^11^; Harun Ilhan, MD^12^; Christoph Rischpler, MD^2^; Axel Wetter, MD^13^; Heiko Schoder, MD^14^; Irene A. Burger, MD^15^.

Affiliations:

2 Department of Nuclear Medicine, University Hospital Essen, University of Duisburg-Essen, Essen, Germany.

8 Department of Radiology, Obihiro Kosei Hospital, Obihiro, Japan and Department of Nuclear Medicine, Hokkaido University Graduate School of Medicine, Sapporo, Japan.

9 Department of Theranostics and Nuclear Medicine, St Vincent’'s Hospital, Sydney, Australia.

11 Department of Nuclear Medicine, Aalborg University Hospital, Aalborg, Denmark.

12 Department of Nuclear Medicine, Ludwig-Maximilians-University Munich, Munich, Germany.

13 Department of Diagnostic and Interventional Radiology and Neuroradiology, University of Duisburg-Essen, Essen, Germany.

14 Molecular Imaging and Therapy Service, Department of Radiology, Memorial Sloan Kettering Cancer Center, New York, USA.

15 Department of Nuclear Medicine, University Hospital Zürich, University of Zürich, Switzerland.
