# Supplementary material for: False positive PSMA PET for tumor remnants in the irradiated prostate and other interpretation pitfalls in a prospective multi-center trial
Source: Eur J Nucl Med Mol Imaging. 2020 Aug 17;48(2):501–8. doi: 10.1007/s00259-020-04945-1 (PMC7835157; doi:10.1007/s00259-020-04945-1)

**False Positive PSMA PET for Tumor Remnants in the Irradiated Prostate and other Interpretation Pitfalls in a Prospective Multi-Center Trial**

**SUPPLEMENTAL MATERIAL**

**Supplemental Table 1. PET/CT and PET/MRI Imaging Parameters.** Abbreviations: ADT, androgen deprivation therapy; SD, standard deviation.

|  |  | **PET/CT (N=443)** | |  | **PET/MRI (N=192)** | |
| --- | --- | --- | --- | --- | --- | --- |
| **Parameter** |  | **N** | **(%)** |  | **N** | **(%)** |
| Injected activity (mCi) | Median (SD) | 5.1 | (0.9) |  | 5.4 | (1.3) |
| Uptake time (hh:mm) | Median (SD) | 01:01 | (00:08) |  | 01:14 | (00:15) |
| Furosemide given |  | 394 | 89 |  | 192 | 100 |
| Contrast given |  | 422 | 95 |  | 188 | 98 |
| PET under ADT |  | 60 | 14 |  | 17 | 9 |

**Supplemental Table 2. [^68^Ga]Ga-PSMA-11 PET/CT and PET/MRI False Negative Findings.** Findings are illustrated in Supplemental Figure 2A (CT) and B (MRI). miTNM stage in accordance with PROMISE [8]. Abbreviations: FN, false negative; Tr, prostate bed; N1, pelvic nodes; M1, extrapelvic.

| **Case No** | **PSA (ng/mL)** | **Time from initial therapy (years)** | **Subregion** | **N false readers** | **CT/MRI** | **Visual PET uptake (SUV_max_)** | **Validation triggered by** | **Details** |
| --- | --- | --- | --- | --- | --- | --- | --- | --- |
| CT FN 1 | 0.3 | 8 | Tr | 3 | no lesion | no (2.2) | clinical suspicion | adjacent bladder/urine uptake |
| CT FN 2 | 5.1 | 3 | Tr (Left seminal vesicle) | 2 | no lesion | intermediate (7.2) | PET local read | adjacent bladder/urine uptake |
| CT FN 3 | 0.2 | 5 | Tr | 2 | no lesion | intermediate (5.5) | PET local read | adjacent rectum uptake |
| CT FN 4 | 0.2 | 2 | N1 (mesorectal) | 3 | round shaped 5 mm node | no (1.7) | CT local read | small metastasis |
| CT FN 5 | 2.4 | 7 | Tr (Left seminal vesicle) | 2 | no lesion | intermediate (3.3) | PET local read | small metastasis |
| CT FN 6 | 2.7 | 14 | M1c (lung) | 2 | 13 mm | no (2.4) | CT local read | partial volume, respiratory movement |
| MRI FN 1 | 2.9 | 1 | Tr | 3 | thickening of bladder wall | no (1.8) | MRI local read | adjacent bladder/urine uptake |
| MRI FN 2 | 4.3 | 8 | M1a (retroperitoneal) | 3 | no lesion | low (4.5) | PET local read | small metastasis |

**Supplemental Figure 1. [^68^Ga]Ga-PSMA-11 PET/CT (A) and PET/MRI (B) False Positive (FP) Findings.**


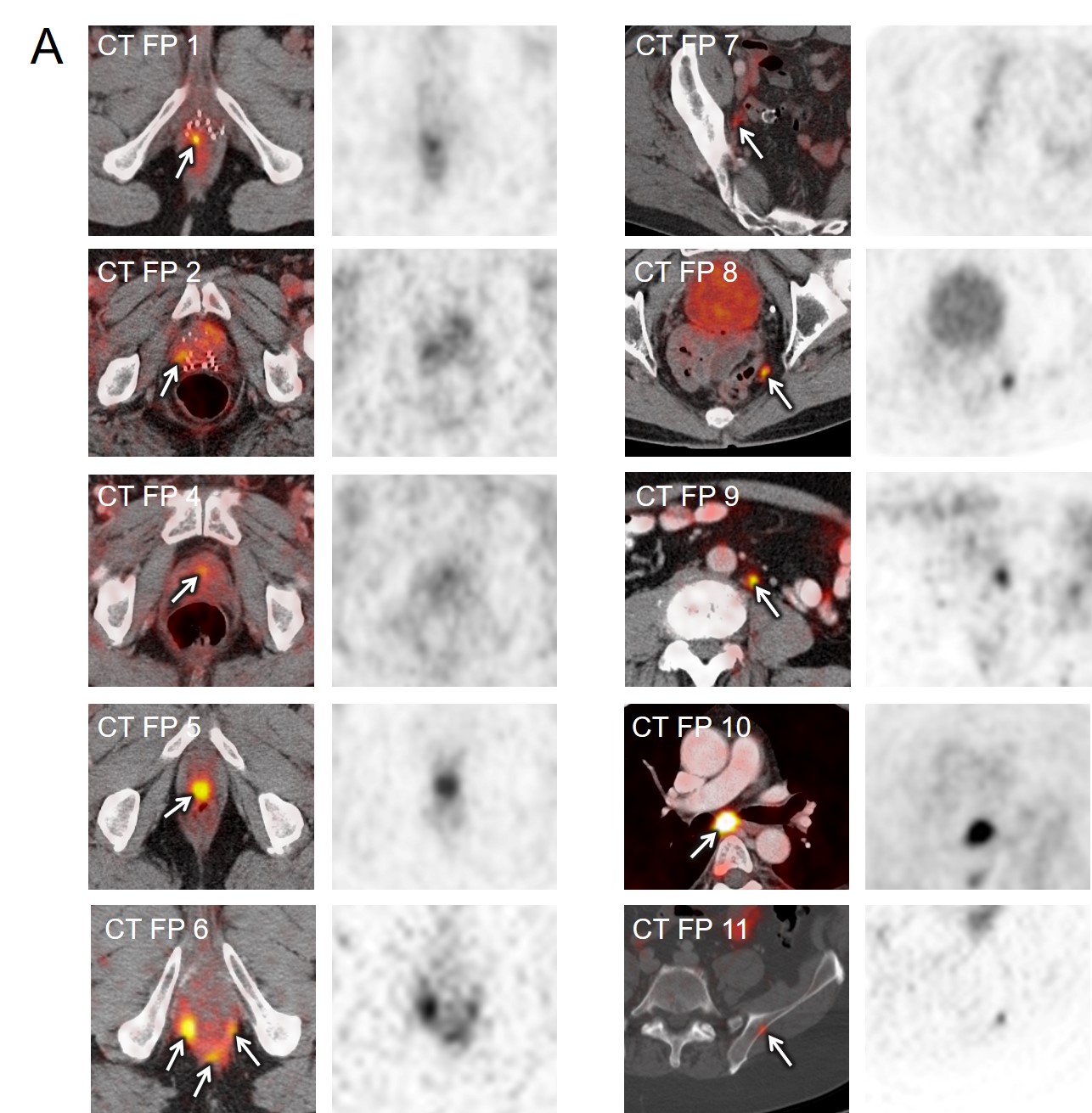


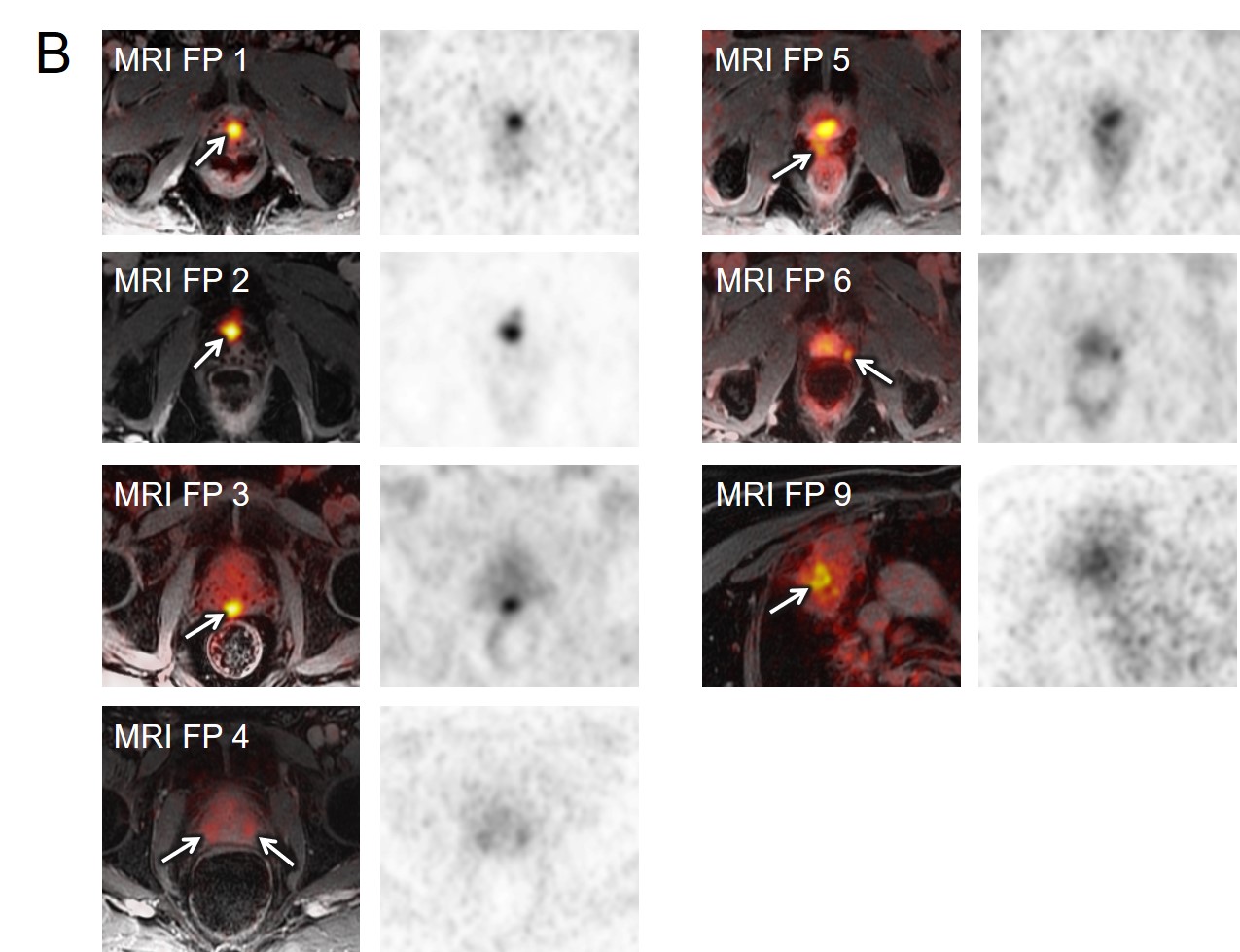


**Supplemental Figure 2. [^68^Ga]Ga-PSMA-11 PET/CT (A) and PET/MRI (B) False Negative (FN) Findings.**


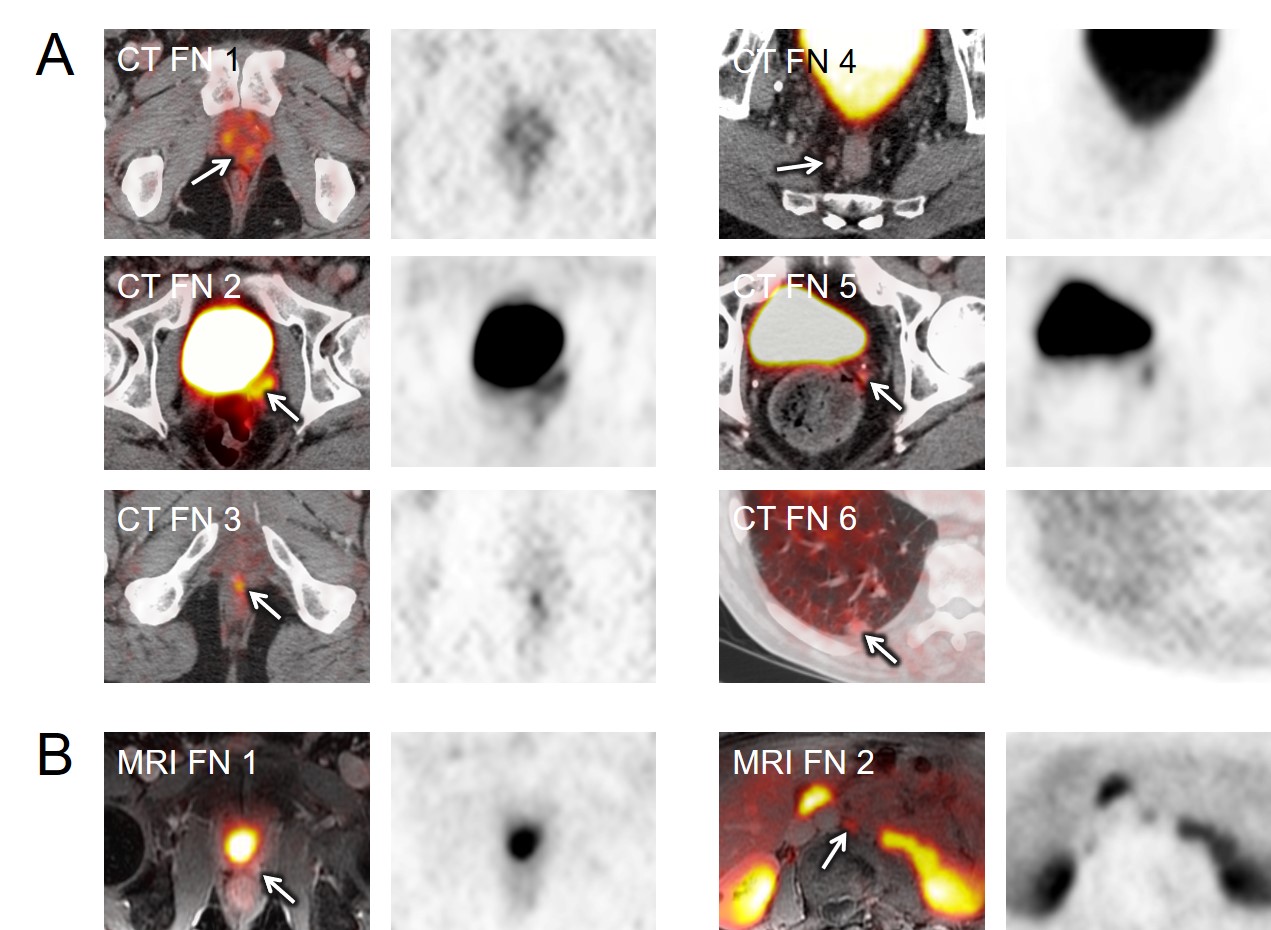

Supplement: Supplementary file 1 — (DOCX 641 kb) [file 259_2020_4945_MOESM1_ESM.docx]
